# A simulation test of the prediction that density-dependent dispersal promotes female-biased sex allocation in viscous populations

**DOI:** 10.17912/micropub.biology.000821

**Published:** 2023-07-11

**Authors:** Chedhawat Chokechaipaisarn, Andy Gardner

**Affiliations:** 1 School of Biology, University of St Andrews, St Andrews, Scotland, United Kingdom

## Abstract

A classic result of sex-allocation theory is that the sex ratio is predicted to be invariant with respect to the rate of dispersal. However, a recent mathematical analysis has suggested that if individuals are able to adjust their probability of dispersal according to the local density of their neighbourhood, then a lower rate of dispersal will be associated with greater female-bias. Here, we perform a computer simulation test of this prediction. Our simulation data provide strong qualitative support for the prediction, and a Monte Carlo randomization test of significance allows us to reject the null hypothesis of the invariance relationship.

**Figure 1.**
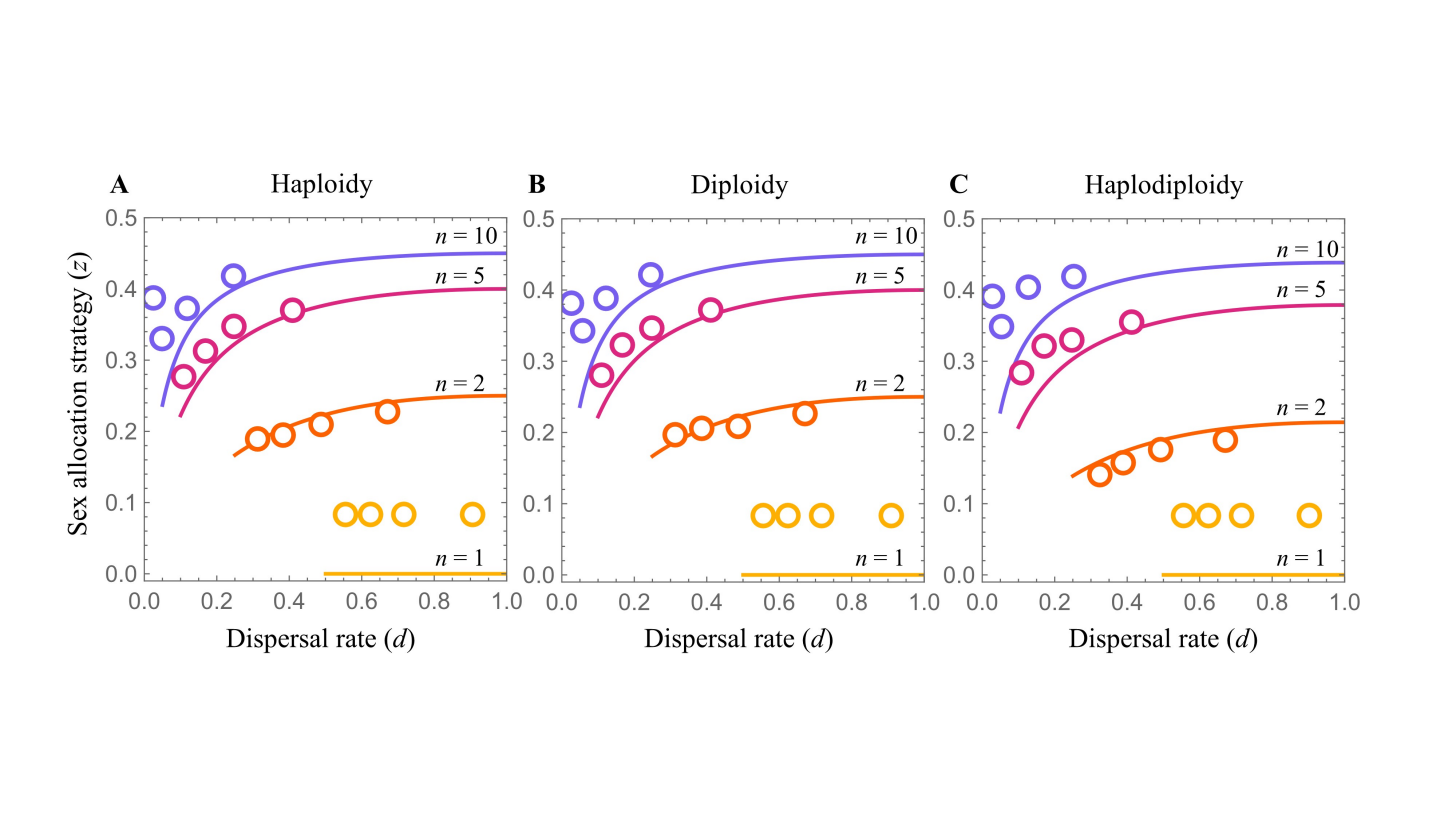
Density-dependent dispersal promotes female-biased sex allocation in viscous populations. There is qualitative agreement between the simulation data (discs) and the predictions of Chokechaipaisarn & Gardner’s (2022) mathematical analysis (lines), under haploidy (A), diploidy (B), and haplodiploidy (C). Results are given for sex allocation ( *z* , the proportional investment into sons) for a range of foundress numbers ( *n* = 1, 2, 5, and 10) and variation in dispersal rate is induced by varying the cost of dispersal (0 < *c* < 1 for the mathematical predictions and *c* = 0.2, 0.4, 0.6, and 0.8 for the simulations). See *Methods* for full details.

## Description

Sex allocation—the apportionment of reproductive resources between sons versus daughters—is the focus of a highly successful programme of research within evolutionary biology and provides among the best quantitative evidence for precision Darwinian adaptation (West 2009) . A particularly productive avenue of study considers competition among genetically-related males for mating opportunities, which promotes female bias (Hamilton 1967) . From this topic emerges the Bulmer-Frank invariant (Bulmer 1986; Frank 1986) , which concerns how a reduced rate of dispersal of mated daughters has two opposing effects on sex allocation that, in the simplest structured-population setting, cancel each other out. First, a lower rate of dispersal of mated daughters increases relatedness within mating groups, which tends to promote female bias. Second, a lower rate of dispersal of mated daughters intensifies local competition for resources, which tends to inhibit female bias. The cancellation holds exactly under haploid and diploid modes of genetic inheritance (Frank 1986; Gardner et al. 2009) , and approximately under haplodiploidy (Taylor 1988) .

However, a recent mathematical analysis has clarified that the Bulmer-Frank invariant result rests upon the assumption that dispersal is density-independent, and has suggested that if mated daughters are able to adjust their probability of dispersal according to the local density of daughters in their neighbourhood then they are favoured to do so and the resulting density-dependent dispersal relaxes local competition for resources and hence alleviates the inhibitory effect of reduced dispersal on female-biased sex allocation (Chokechaipaisarn & Gardner 2022) . Specifically, according to the “constant non-disperser” principle (Crespi & Taylor 1990) , a daughter’s probability of dispersal is expected to increase with the local density of daughters in such a way that all neighbourhoods have the same absolute number of non-dispersing daughters, with all daughters in excess of this number dispersing, which serves to completely abolish the local resource competition effect (Chokechaipaisarn & Gardner 2022; cf Kanwal & Gardner 2022) . This yields the prediction that an overall lower rate of dispersal is associated with greater female-bias in sex allocation under haploid, diploid and haplodiploid modes of inheritance ( Figure 1, solid lines ).

Here, we perform a computer-simulation test of the prediction that enabling mated daughters to adjust their dispersal behaviour according to the local density of daughters will promote female-biased sex allocation in viscous populations, under haploidy, diploidy and haplodiploidy ( Figure 1, discs ; see *Methods* for full details). We consider a range of group sizes ( *n* = 1, 2, 5 and 10 foundresses) and mortality costs of dispersal ( *c* = 0.2, 0.4, 0.6 and 0.8), and allow dispersal and sex allocation to co-evolve. We assume that each mated daughter has a dispersal reaction norm that is a linear function of local density, and allow the intercept and slope of this reaction norm to evolve. For each treatment, we report the overall rate of dispersal and sex allocation within the simulated population after 50,000 generations of evolution.

Overall, the simulated data are well aligned with the analytical predictions ( Figure 1 ). However, there are two exceptions. First, across all costs of dispersal ( *c* = 0.2, 0.4, 0.6 and 0.8) and all modes of genetic inheritance (haploidy, diploidy and haplodiploidy), sex allocation—defined as the proportional investment into sons—observed in the single-foundress ( *n* = 1) simulation treatment is substantially higher than predicted. This discrepancy is expected, as the prediction is more specifically that a mother should produce only as many sons as are required to ensure that all of her daughters are mated, and in the mathematical model the assumption of very large brood sizes (and unlimited male fecundity) results in an unbeatable sex ratio that is very close to zero, whereas in the simulation model the necessary assumption of finite (and, indeed, relatively small) brood sizes results in a minimum viable sex allocation that is substantially greater than zero (specifically, *z* = 1/12 = 0.083 as each mother produces 12 offspring in the *n* = 1 treatment; see *Methods* ).

Second, the fit between predicted and observed sex allocation is relatively poorer when the rate of dispersal is low (left hand side of each panel in Figure 1 ). This owes to a substantial proportion of groups being of sufficiently low density that even if no daughter disperses from the group the number of non-dispersers still falls below the “constant non-disperser” threshold, such that a greater investment into females does not result in a greater rate of dispersal and hence leads to an intensification of local competition for resources—a complexity that does not arise in the mathematical model, which assumes only vanishingly small differences in local density. For example, in the *n* = 10, *c* = 0.8 simulation treatments, approximately 30% of patches are too small to produce the required number of non-dispersing individuals, substantially reducing the scope for competition alleviation. Implementing larger brood sizes would be expected to bring the simulation results more closely in line with the theoretical predictions as this would reduce stochastic variation in relative density across groups.

Nevertheless, the simulations do provide strong support for the qualitative prediction that enabling mated daughters to adjust their dispersal behaviour according to local density promotes the evolution of female-biased sex allocation in viscous populations. For all foundress-number treatments—except for *n* = 1, in which there is predicted to be no effect of dispersal on sex allocation—and under all modes of genetic inheritance, there is a clear trend for lower overall rates of dispersal—induced by increasing the mortality cost of dispersal—to be associated with a greater degree of female bias. For haploidy and diploidy, under which the Bulmer-Frank invariant is expected to apply exactly, we can reject the null hypothesis of there being no relationship between dispersal and sex allocation by means of a Monte Carlo randomization test ( *P* < 0.001; see *Statistical Analysis* ). For haplodiploidy, the Bulmer-Frank invariance is not predicted to hold exactly, and so it is not meaningful to apply the statistical test in this case.

## Methods

We generated simulated populations subdivided into 1000 discrete patches with number of foundresses *n* = 1, 2, 5, and 10 per patch. For each scenario, we ran simulations with four different values of cost of dispersal *c* = 0.2, 0.4, 0.6 and 0.8, giving a total of 4 x 4 = 16 separate simulations for each of the three modes of genetic inheritance: haploidy, diploidy and haplodiploidy. Each individual’s genome consists of three loci: the first locus determining their sex allocation strategy, the second locus controlling their probability of dispersing from a patch of zero density, and the third locus controlling their probability of dispersing from a patch of maximum density. The genes at the first locus are allowed to take any values between negative and positive infinity, and the value of a haploid mother’s single gene or the average value of a diploid mother’s two genes at this locus determines the independent probability that any given one of her offspring is male. Here, a sex allocation strategy of less than zero is interpreted as meaning that all of her offspring are female, and a sex allocation strategy of more than one is interpreted as meaning that all of her offspring are male. The genes at the second and third loci are also allowed to take any values between negative and positive infinity, and the value of a haploid female’s single gene or the average value of a diploid female’s two genes at the corresponding locus determines her probability of dispersing under zero density or maximum density conditions; her probability of dispersing under intermediate density is given by a linear reaction norm connecting these two end points. Here, a dispersal probability of less than zero is interpreted as meaning that the female does not disperse, and a dispersal probability of more than one is interpreted as meaning that she does disperse.

At initialization we set the allelic value of every gene at the first locus to the unbeatable sex-allocation strategy obtained in Chokechaipaisarn & Gardner’s (2022) density-independent dispersal analysis, i.e. their equation (2.1) for haploidy and diploidy, and their equation (2.3) for haplodiploidy, and we set the allelic value of every gene at the second and third loci to the unbeatable dispersal strategy obtained in Chokechaipaisarn & Gardner’s (2022) density-independent dispersal analysis, i.e. 2/(1+2 *cn* +(1+4 *n* ( *n* -1) *c* ^2^ ) ^1/2^ ). Each mother produces 10 offspring, with the sex of each offspring being determined by the mother’s sex allocation genes. To ensure at least there is one mated daughter per patch, we randomly assigned one mother per patch to produce an additional son and we randomly assigned one mother per patch to produce an additional daughter. Offspring inherit genes from their parents according to the haploid, diploid or haplodiploid scheme, with each gene having a probability 0.01 of mutating, in which case its allelic value increases by a random increment sampled from a normal distribution with mean zero and standard deviation 0.1.

A generational step proceeds as follows: each mother produces offspring and dies, then random mating take place among offspring within their natal patch, where each female can mate once with a random male and each male can mate potentially numerous times. After mating, all males die, and mated daughters assess the local population density and either remain in their natal patch or else attempt to disperse to a new, randomly chosen patch, according to the alleles at their second and third loci. With probability 1- *c* a daughter who attempts dispersal successfully relocates to her destination patch and with probability *c* she dies during the attempt. following dispersal, *n* daughters are chosen at random on each patch to be the mothers of the next generation. This generational step was repeated for 100,000 generations, for each of 16 simulation runs for each mode of genetic inheritance. For each simulation, the population sex ratio and the proportion of individuals attempting dispersal are averaged over the final 50,000 generations, resulting a single data point (disc) in Figure 1 . A *Mathematica* notebook (and a PDF version of the same) containing the simulation code is provided as *Extended data* .

**Statistical Analysis**

A Monte Carlo randomization test was performed by aggregating the 24 data points from the haploidy and diploidy treatments, excluding the *n* = 1 data. A simple mixed-effects linear regression model, with a random slope for *n* , was used to determine the regression coefficient of the actual data and 100,000 randomizations. The *P* -value was calculated as *P =* ( *R+* 1)/( *N+* 1), where *N* is the total number of randomizations and *R* is the number of randomizations that have regression coefficients that are equal to or larger than that of the actual data.

## Extended Data

Description: Mathematica notebook containing simulation code and statistical analysis. Resource Type: Model. DOI: 10.22002/17pny-n3v48

Description: PDF file of Mathematica notebook containing simulation code and statistical analysis. Resource Type: Model. DOI: 10.22002/rnzse-p0686
